# Au Nanoparticles Immobilized on Honeycomb-Like Polymeric Films for Surface-Enhanced Raman Scattering (SERS) Detection

**DOI:** 10.3390/polym9030093

**Published:** 2017-03-06

**Authors:** Chia-Yen Chiang, Ting-Yu Liu, Yu-An Su, Chien-Hsin Wu, Yu-Wei Cheng, Ho-Wen Cheng, Ru-Jong Jeng

**Affiliations:** 1 Institute of Polymer Science and Engineering, National Taiwan University, Taipei 10617, Taiwan; r04549007@ntu.edu.tw (C.-Y.C.); kizukisu@gmail.com (Y.-A.S.); chienhsinwu@hotmail.com (C.-H.W.); louischengblue@gmail.com (Y.-W.C.); 2 Department of Materials Engineering, Ming Chi University of Technology, New Taipei City 24301, Taiwan; 3 Institute of Atomic and Molecular Sciences, Academia Sinica, Taipei 10617, Taiwan; r01222047@ntu.edu.tw

**Keywords:** surface-enhanced Raman scattering (SERS), breath figure, honeycomb-like polymeric films, gold nanoparticles, hot-spots resonance effects

## Abstract

We have successfully developed novel surface-enhanced Raman scattering (SERS) substrates with three-dimensional (3D) porous structures for effectively improving the sensitivity and reproducibility of SERS, which can rapidly detect small molecules (rhodamine 6G as an example). Periodical arrays of the honeycomb-like substrates were fabricated by self-assembling polyurethane-*co*-azetidine-2,4-dione (PU-PAZ) polymers. PU-PAZ comprising amphiphilic dendrons could stabilize the phase separation between the water droplets and polymer solution, and then organize into regular porous structures during the breath figure method. Subsequently, SERS substrates were fabricated by immobilizing gold nanoparticles (AuNPs) onto the honeycomb-like films with various 3D porous structures, controlled by the different PU-PAZ concentrations and relative humidities. Results show that surface enhancement factors of honeycomb-like substrates were 20 times higher than that of flat-film substrates (control group) due to enormous hot-spots resonance effects by the 3D porous structure, verified through Raman mapping at various positions of the *z*-axis. Furthermore, the particle size effects were evaluated by immobilized 12 and 67 nm of AuNPs on the honeycomb-like substrates, indicating larger AuNPs could induce more pronounced hot-spots effects. The generation of hot-spots resonance to enhance Raman intensity is strongly dependent on the diameter of AuNPs and the pore size of the honeycomb-like and 3D porous substrates for label-free and rapid SERS detection.

## 1. Introduction

Surface-enhanced Raman scattering (SERS) has recently shown tremendous potential in detecting a wide range of trace-level analytes, from single functional molecules to complex biomaterials [1,2,3,4,5,6]. The SERS effect improves the intrinsic limitation of a weak Raman scattering signal when detecting a low concentration of targeting samples. In comparison, the Raman spectrum is a collection of inelastic scattering photons from measured molecules, which provides specific vibrational fingerprints of chemical structures. Nevertheless, these small quantities of scattered photons are difficult to detect. Yet, Fleischmann et al. [7] discovered the Raman intensity of pyridine was powerfully amplified when absorbing the compound on roughed silver substrates, and thus initiated the Raman detection of this SERS phenomenon. Many researchers have reported that the SERS phenomenon in specific nanostructured substrates contributed to the quantities of hot spots between plasmonic nanoparticles [8,9,10]. As a result, SERS has been widely applied in identifying chemical molecules, biological materials, as well as environmental/water pollutants in a facile manner [4,11,12,13,14].

Diverse SERS-active substrates with surface roughness at the nanoscale have been fabricated in the recent decade [1,15,16,17,18,19]. Most of all, SERS substrates have been made by noble metal nanoparticles, especially gold nanoparticles (AuNPs) and silver nanoparticles (AgNPs) [20,21,22,23,24]. Although AgNPs are capable of supplying intense SERS signals, AuNPs provide better reproducible SERS signals with less toxicity and a simple functionalization strategy. The optimal hot spots of SERS substrates can be modulated by controlling the concentration and particle size of the noble metal nanoparticles in the colloidal solutions [25,26]. Moreover, it is well known that the aggregated AuNPs can produce enormous enhancement. Yet it is difficult to manipulate the uniform aggregation over a large area. Thus, there have been alternative substrates using anisotropic AuNPs [19].

In addition, strategies for developing highly homogenous nanoparticle arrays to improve the reproducibility of SERS substrates has been thoroughly investigated [27,28,29,30]. To produce nano-level periodical roughness on the surface, lithography is an often used approach [31]. Uniform SERS nanoparticle arrays have been developed in our previous work, which immobilized silver nanoparticles in the homogenous arrays of anodic aluminum oxide (AAO) nano-channels by electro-chemical lithography methods [8]. Furthermore, the breath figure method (BF method) is another process used to achieve ordered arrays via molecular assembly, which is considered low-cost, less time consuming, and easy to implement [32,33,34,35]. Hao and his coworkers [36,37] first came up with honeycomb-patterned films (HPFs) substrates made up of AuNPs, and revealed that these substrates full of surface roughness at the nanoscale could somehow solve the problem of extremely weak Raman signals of analytes. Afterwards, Wan et al. [38] produced a AgNP-containing honeycomb-like SERS substrate and demonstrated great enhancement of Raman signals with a surface enhancement factor (SEF) of 4 × 10^8^. In addition, Wang et al. [39] illustrated a honeycomb porous substrate featuring selectivity of AgNPs to obtain a controllable SERS effect. Based on the above, both the honeycomb porous structure and flat-film substrates with highly ordered nanoparticles arrays were able to enhance the SERS effect, with huge enhancement in the former. However, the detailed causes for such enhanced phenomena were not revealed. Therefore, we set out to investigate the mechanism of Raman enhancement by hot-spots resonance effects in the three-dimensional (3D) honeycomb porous structure along with the flat-film substrates as a control [23,40,41].

**Scheme 1:**
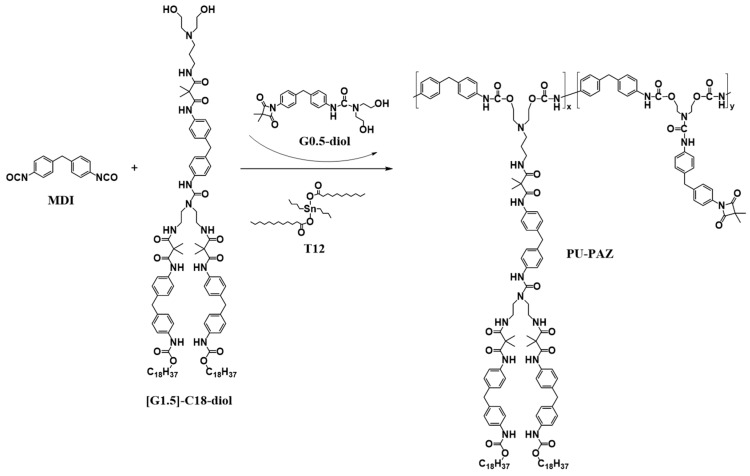
Synthesis of PU-PAZ polymers.

Herein, we fabricated novel SERS-active and honeycomb-like nanoparticle arrays by a BF process with a dendritic polymer, polyurethane-*co*-azetidine-2,4-dione (PU-PAZ; Scheme 1), and then immobilized AuNPs on the honeycomb-patterned substrates for SERS detection [42]. The formation of honeycomb-like structures assisted by polymers has been extensively investigated [43,44,45,46,47]. In particular, we have synthesized several dendritic polymers via a convergent route [48,49,50,51,52]. These dendritic polymers exhibiting amphiphilic properties could induce self-assembly, and achieve periodical honeycomb-patterned porosities through the BF method [33,35,42,53,54].

This study provides a clear picture of hot-spots effects in 3D honeycomb-like nanoparticle arrays. Apart from that, the sensitivity and reproducibility of SERS substrates were also investigated in great detail by Raman spectroscopy and mapping. The characteristics of the honeycomb-like polymeric films and AuNPs were evaluated by scanning electron microscope (SEM), transmission electron microscope (TEM), UV–Vis spectrophotometer, dynamic light scattering (DLS), and electron spectroscopy for chemical analysis (ESCA).

## 2. Materials and Methods

### 2.1. Materials

Methylene di-p-phenyl diisocyanate (MDI), isobutyryl chloride, triethylamine (TEA), stearyl alcohol, diethylenetriamine (DETA), xylene, cyclohexane, methanol, *N*,*N*-dimethylformamide (DMF), tetrahydrofuran (THF), dimethylsulfoxide (DMSO), chloroform, toluene, Gold(III) chloride trihydrate, trisodium citrate dihydrate (99%), and rhodamine 6G (R6G) were purchased from Sigma-Aldrich Corp. (St. Louis, MO, USA) and Acros Organics (Ozaukee County, WI, USA). *N*-(3-aminopropyl) diethanolamine (APDEA) and dibutyltin dilaurate (DBTDL, or T12) were purchased from TLC Pharmaceutical Standards Ltd. (Aurora, ON, Canada). Silver nitrate (99.8%) was purchased from SHOWA Corp. (Gyoda, Japan). The deionized water from an ELGA PURELAB Classic system (ELGA LabWater Veolia Water Technologies, Sanchung, New Taipei City, Taiwan) with resistivity higher than 18.2 MΩ was used in this study.

### 2.2. Synthesis of Gold Nanoparticles (AuNPs)

Before synthesizing gold nanoparticles, all glassware and stir bars should be first cleaned with aqua regia solution (HCl/HNO_3_, volume ration, 3:1) to make sure no metal ions remain, and should then be washed with a large amount of deionized water.

Spherical AuNPs were synthesized by an improved Turkevich method [55,56] which reduced tetrachloroauric(III) acid with trisodium citrate as described in the following. First, 0.5 wt % HAuCl_4_, 1 wt % trisodium citrate, and 0.1 wt % AgNO_3_ were prepared in aqueous solution and these three solutions were stored at 4 °C. Subsequently, a solution was prepared by mixing 85 μL AgNO_3_ and 2 mL HAuCl_4_, and then various amounts of trisodium citrate aqueous solution (0.4 mL or 3 mL) were added to the solution, respectively. This solution was further diluted with deionized water to a total volume of 5 mL. This solution was subsequently added to boiling deionized water (95 mL) with active stirring in a 250 mL two-neck flask. The solution color turned from light yellow, to purple, to ruby red. It is important to note that various sizes of AuNPs were obtained after the one-hour reaction, dependent on the addition volume of the trisodium citrate solution.

### 2.3. Preparation of PU-PAZ Polymers

The synthesis for the PU-PAZ polymers is described in our previous work [42]. As shown in Scheme 1, [G-1.5]-C18-diol and G0.5-diol were first prepared from a dual-functional compound, 4-isocyanato-4-(3,3-dimethyl-2,4-dioxo-acetidino) diphenylmethane (IDD). The IDD compound derived from MDI is featured for its isocyanate with high reactivity toward active hydrogens, and azetidine-2,4-dione with selective reactivity toward aliphatic primary amines. The PU-PAZ was polymerized based on MDI, [G-1.5]-C18-diol, and G0.5-diol by a typical two-step process for the preparation of polyurethanes in DMF under N_2_ atmosphere (Scheme 1).

### 2.4. Preparation of PU-PAZ Honeycomb-Like Polymeric and Flat Films

Honeycomb-like polymeric films were processed using various concentrations of polymer solution under a specific range of relative humidity (RH) through the BF technique. An amount of PU-PAZ chloroform solution (50 μL) was spread onto a 1 cm^2^ silicon wafer at room temperature under high RH. The temperature and RH was maintained constant in an environment chamber using a KCL-2000A (EYELA, Bunkyo-ku, Tokyo, Japan) which can control the temperature and relative humidity. Honeycomb-like films based on PU-PAZ were obtained after complete evaporation of chloroform and water droplets. The morphology was controlled by the concentration of PU-PAZ ranging from 5 mg/mL to 20 mg/mL, and the RH from 50% to 95%.

For the sake of comparison, the flat-film substrates (FFSs) without honeycomb-like pores were fabricated by dissolving PU-PAZ in toluene with a concentration of 20 mg/mL. This polymer solution (50 μL) was dropped onto a 1 cm^2^ silicon wafer and placed in vacuum at room temperature to remove toluene.

### 2.5. Preparation of SERS Substrates

**Scheme 2:**
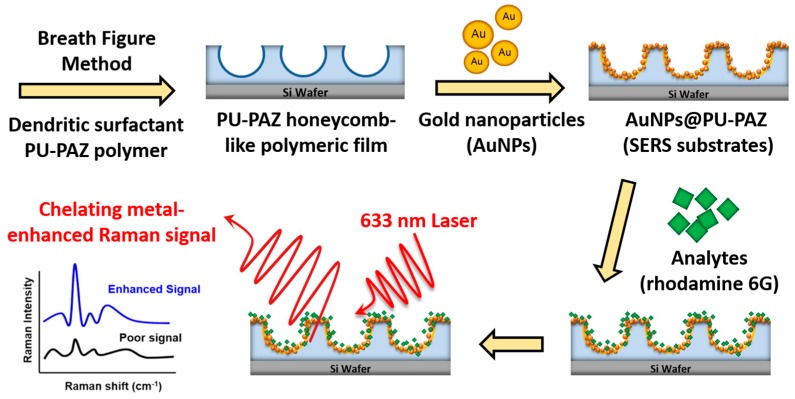
Diagram of the honeycomb-like surface-enhanced Raman scattering (SERS) substrates fabrication.

In order to prepare the polymer nanocomposite SERS substrates, the PU-PAZ honeycomb-like polymeric films were immersed in 2 mL of as-prepared aqueous solution with different sizes of AuNPs (12 and 67 nm). After 6 h, the polymeric films on the silicon wafer were taken out of the solution and rinsed with deionized water thoroughly several times to obtain the final SERS substrates. Afterwards, 5 μL of a 10^−6^ M rhodamine 6G (R6G) aqueous solution was dropped on various honeycomb-like polymeric SERS substrates (1 cm^2^) for further SERS measurements. The process of preparing honeycomb-like SERS substrates is shown in Scheme 2. The control group of PU-PAZ flat films were also prepared in the same manner.

### 2.6. Characterization

#### 2.6.1. Characterization of AuNPs

UV–Vis absorption spectra were recorded using an UV–Vis spectrophotometer in the wavelength ranging from 400 to 800 nm. The distribution of AuNPs with different sizes were characterized by transmission electron microscopy (TEM) and high resolution TEM (HR-TEM) images using a Zeiss EM 912 Omega microscope (Carl Zeiss MicroImaging Inc., Oberkochen, Germany) with 120 and 200 kV acceleration voltages, respectively. The TEM samples were prepared by dropping a 5 μL colloidal solution of AuNPs on a 100 mesh copper grid coated with carbon and dried at room temperature. The average ferret diameters with standard deviations of AuNPs were measured by an open source image processing program, ImageJ [57] (National Institutes of Health (NIH), Bethesda, MD, USA). The determination of hydrodynamic diameters and size distribution of nanoparticles in solution were performed by using dynamic light scattering (DLS) at room temperature with a fixed angle of 90°. The number-average values were used to express the size distributions of AuNPs, which was near what demonstrated in the TEM images. The binding energy (Au 4f) of AuNPs was analyzed by electron spectroscopy using a VG ESCA Scientific Theta Probe (VG Scienta Inc., Newburyport, MA, USA) for chemical analysis.

#### 2.6.2. Characterization of Honeycomb-Like Polymeric Films

The dendrons and PU-PAZ polymers were characterized in the same manner as those in our previous studies [33,35,42,54]. Morphologies of the films were observed with scanning electron microscopy (SEM) using a field-emission SEM (JSM S-6700F, JEOL Inc., Peabody, MA, USA) with 10 kV of electron gun voltage. Before measurement, the films were sputter coated with platinum to attain higher resolution images. The average pore sizes of the polymeric films were also obtained by using ImageJ software for automatic calculations.

### 2.7. Measurement of Surface-Enhance Raman Scattering

An amount of 5 μL of a 10^−6^ M rhodamine 6G (R6G) aqueous solution was dropped on 1 cm^2^ of various SERS substrates, and dried for further SERS measurements. Raman spectrums were measured by LabRAM HR800 Evolution Raman spectroscopy (HORIBA Ltd., Minami-ku, Kyoto, Japan) using a 633 nm solid-state laser light as the excitation source in the range of 600 to 1800 cm^−1^. Typical measurements were under the conditions of 5 s of laser exposure time, 3 times of spectra accumulation, with a 50× of objective lens and 61.3 μW of laser input power. As for two-dimensional (2D) Raman mapping measurements, the exposure time increased to 8 s, with 3 times of data accumulation, through a 100× of objective lens and 4.1 μW of laser input power. To analyze the reproducibility of the SERS substrates, 16 different sites on the substrates were randomly selected to collect Raman spectra. All the measurements were performed at room temperature. Before each experimentation, the Raman shift was calibrated by a signal located at 520 cm^−1^ with absolute intensity from a standard silicon wafer.

## 3. Results and Discussion

### 3.1. Characterization of AuNPs

**Figure 1:**
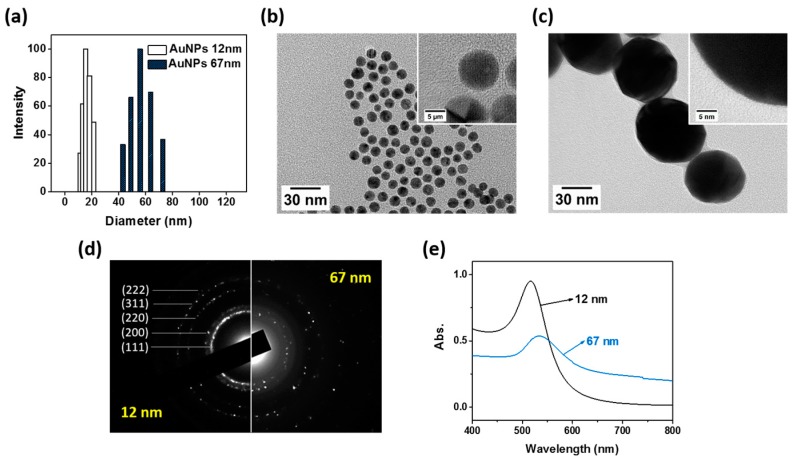
Characteristics of AuNPs (**a**) AuNPs size distribution analyzed by dynamic light scattering (DLS); (**b**) Transmission electron microscope (TEM) images of AuNPs (12 nm); (**c**) TEM images of AuNPs (67 nm); (**d**) Selected area diffraction (SAED) of AuNPs; (**e**) UV–Vis absorption spectra of AuNPs.

**Figure A1:**
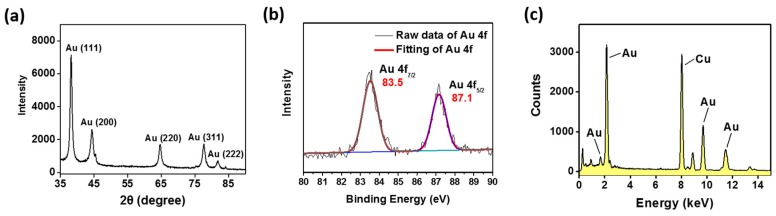
Characteristics of AuNPs. (**a**) X-ray diffraction (XRD) of AuNPs; (**b**) Electron spectroscopy for chemical analysis (ESCA) at Au-4f, with characteristic peaks at 87.1 and 83.5 eV; (**c**) energy dispersive spectrometer (EDS) spectrum confirming elemental gold composition.

AuNPs were fabricated by a modified traditional Turkevich protocol to obtain quasi-spherical AuNPs in aqueous solution with narrow size distribution and uniform shape [55,56]. The diameter of AuNPs decreased from 67.3 ± 3.3 to 11.8 ± 1.3 nm with increasing concentration of the reducing agent from 0.4 to 3 mL, as shown in Figure 1a of the DLS measurement. TEM and HR-TEM images (the insert) also showed the diameters of AuNPs in Figure 1b (12 nm), and Figure 1c (67 nm). Selected area diffraction (SAED) images of 12 and 67 nm Au nanoparticles were illustrated in Figure 1d, showing diffraction planes of (111), (200), (220), (311), and (222). The result is similar to that reported in the literature [58]. The XRD spectrum (Figure A1a) also demonstrated five diffraction peaks, corresponding to the five crystal planes of AuNPs, similar to the SAED results.

Furthermore, the characteristic surface plasmon resonance (SPR) of AuNPs was found to be located at 516 and 534 nm in UV–Visible spectrum (Figure 1e). After calculation by the method provided by Haiss et al. [59], the diameters of AuNPs are similar to those observed from DLS and TEM. The sizes distribution of AuNPs could be well-tuned by the modified traditional Turkevich protocol. This is beneficial for the reproducibility of further SERS substrate development. In addition, two characteristic peaks of AuNPs were found at binding energies of 83.5 eV (Au 4f_7/2_) and 87.1 eV (Au 4f_5/2_) in ESCA (Figure A1b), along with the presence of four X-ray photon peaks of Au in the energy dispersive spectrometer (EDS) spectrum (Figure A1c; Cu signal might come from the copper grids). This corroborates the successful synthesis of AuNPs.

### 3.2. Morphology of Honeycomb-Like Films Based on PU-PAZ

Highly ordered honeycomb-like polymeric films were obtained through the BF method with the dendritic polyurethane, PU-PAZ. The solution of the dendritic surfactant, PU-PAZ, was spread on the silicon wafer in a moist environment. With the rapid evaporation of solvent (CHCl_3_), the surface temperature of the casting solution dropped. Subsequently, water droplets would start to condense on the surface. Afterwards, PU-PAZ could stabilize the interface between the water and polymer solution, arranging water droplets in order. Finally, honeycomb-like structures were obtained as the water evaporated. The sizes and uniformity of honeycomb-like porosities were essentially affected by the parameters such as polymer and solvent type, polymer solution concentration, humidity, and temperature [33,42,60,61,62,63]. Among these parameters, researchers tend to manipulate pore size and the distance of honeycomb-like films via the variations of RH and/or polymer solution concentration [42,64].

**Figure 2:**
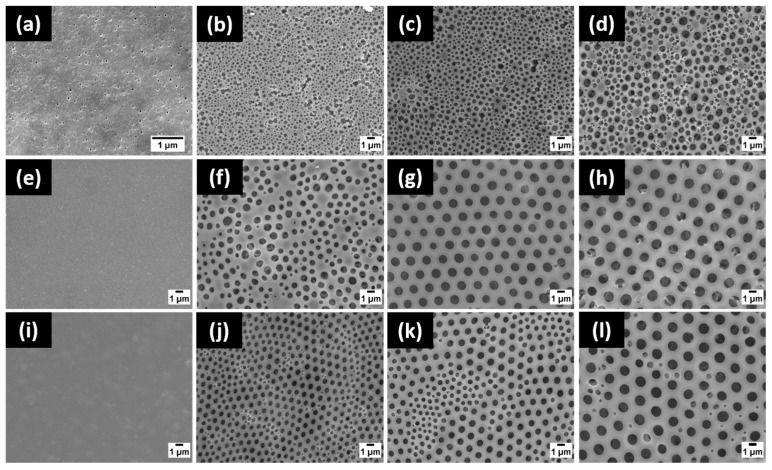
SEM images of honeycomb-like polymeric PU-PAZ films with different concentrations of polymer solution and relative humidity (RH). (**a**) 50%; (**b**) 65%; (**c**) 80%; (**d**) 95% of RH in 5 mg/mL PU-PAZ solution; (**e**) 50%; (**f**) 65%; (**g**) 80%; (**h**) 95% of RH in 10 mg/mL PU-PAZ solution; (**i**) 50%; (**j**) 65%; (**k**) 80%; (**l**) 95% of RH in 20 mg/mL PU-PAZ solution at 25 °C.

Via SEM investigation, different morphological changes of the honeycomb-like polymeric films were observed with various polymer solution concentrations (5–20 mg/mL) and RH (50%–95% with 15% of increment) (Figure 2). The results reveal that the film prepared from 5 mg/mL of polymer solution was full of broken and disordered pores due to the lack of surfactants to stabilize the interface between the water droplet and polymer solution. On the contrary, highly regular honeycomb-like films could be fabricated from a higher concentration of polymer solution (from 10 to 20 mg/mL), since the aggregation of water droplets was prevented by the sufficient repulsion force from PU-PAZ. Moreover, average diameter of the pores decreased due to fast stabilization and higher viscosity of the solution to slow down the growth of water droplets, leading to smaller pore sizes after water evaporation. Apart from that, the film exhibited no pore or few irregular pores when the RH was lower than 50% (Figure 2a,e,i). This is because few water droplets condensed on the surface of the polymer solution. As RH increased from 65% to 95%, the degree of water condensation increased and consequently water droplets became bigger, leading to the larger honeycomb-like pores (Figure 2b–d,f–h,j–l). Moreover, the films exhibited not only larger pore sizes, but also a more even distribution of pores under higher RH. To summarize, the optimized honeycomb-like films featuring periodical distribution of pores can be achieved for the polymer solution with the concentration of 10 mg/mL under higher RH (80% in Figure 2g, 95% in Figure 2h).

Both films fabricated under 80% and 95% RH exhibited highly regular honeycomb-like arrays. In particular, an even better morphology was obtained for the film prepared under higher RH. Therefore, we chose two honeycomb-like films (prepared under 65% and 95% RH) with distinct morphologies for further investigation. Table 1 shows the pore sizes for the honeycomb-like and flat-film substrates prepared from polymer concentrations at 5, 10, and 20 mg/mL under RH at 65% and 95% (simplified by lower (L) and higher (H) RH for 65% and 95%, respectively). For the sample code, honeycomb substrate is assigned as “HS”, whereas the number followed after “HS” represents the polymer concentration. The listed pore sizes were calculated with imaging software analysis. Through the adjustment of the polymer concentration and RH, the pore sizes could be tailored from 0.5 to 1.5 μm as evidenced by the SEM images (Figure 2).

### 3.3. Hot-Spots Effect in the 3D Honeycomb-Like Polymeric SERS Substrate

**Figure 3:**
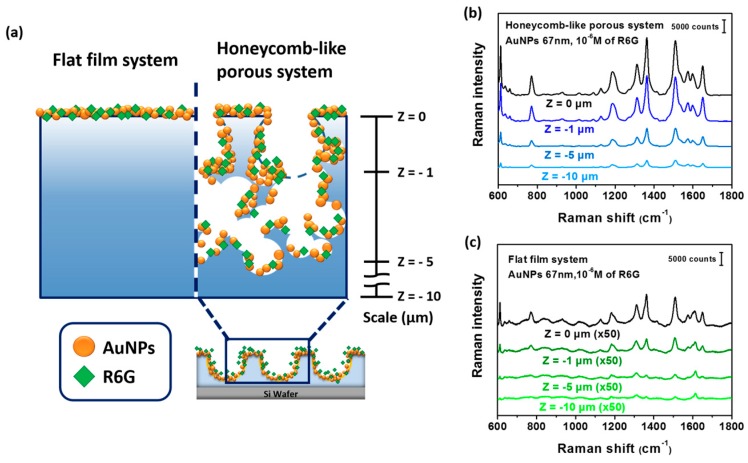
(**a**) Schematic of the SERS mechanism between honeycomb-structured and flat-film substrates; SERS spectra of (**b**) honeycomb-structured and (**c**) flat-film substrates at different vertical positions labeled in the *z*-axis (*z* = 0, −1, −5, −10 μm). The concentration of analytes (R6G) is 10^−6^ M, and the size of AuNPs is 67 nm.

Figure 3a illustrates the schematic diagram of hot-spots resonance effects for the AuNPs arrays of honeycomb-like polymeric SERS substrates (right-hand side), and flat-film SERS substrates (left-hand side). Honeycomb-like polymeric film and flat-film substrates were immersed into the as-prepared AuNPs aqueous solution to form Au nanoparticle arrays of SERS substrates with rhodamine 6G (R6G, 10^−6^ M) used as the target analytes. Since the honeycomb-like substrates possessed multilayer porosity, a majority of AuNPs were physically adsorbed not only on the surface, but also on the walls of the pores inside the film. With that in mind, laser excitation would bring about surface plasmon resonances in AuNP arrays of honeycomb-like polymeric SERS substrates. As a result, the Raman signals of analytes multiplied and produced enormous SERS enhancement, elucidating the multiplied hot-spots effect [40,41].

In order to analyze Raman signals at different positions of the *z*-axis, a confocal Raman microscope was used to precisely control the scanning depth without any damage. As illustrated in Figure 3b,c, the characteristic peaks conform to the exact Raman signals of R6G molecules. The peak at 613 cm^−1^ represented C–C–C ring in-plane bending, while the strong C–H in-plane bending peak was located at 1360 cm^−1^. Other intense Raman signals could be observed at 1510 and 1652 cm^−1^, contributed by aromatic CC stretching [7]. As the position of laser scanning depth increased (0 to −10 μm), the relatively weak Raman intensity indicates poor signal enhancement at deeper layers. Even so, the SERS effect in the honeycomb system was still greater than that in the flat-film system. Due to the presence of multilayered honeycomb-like porous structures, the laser beam (633 nm) was able to not only spot the surface-immobilized AuNPs, but also penetrate to the AuNPs located on the contours of deeper pores. The penetrating laser beam went through “repeating irradiations and reflections” inside the multilayered honeycomb-like pores, and finally returned to the surface, generating the so-called hot-spots resonance effects in the 3D structure. As a consequence, a tremendous increment of Raman intensity could be provided through a collection of localized surface plasmon resonance (LSPR) generated by the attached AuNPs on and beneath the surface, when compared with the flat film. Furthermore, it was reported that the aggregation of plasmonic nanoparticles among adjacent nanoparticles would increase hot spots formation [65]. In our system, this might be attributed to enhancing Raman scattering owing to hot-spots resonance effects of AuNPs clusters in the 3D honeycomb-like porous structure. With the accumulation of enhanced Raman signals, an intense SERS effect was observed in the honeycomb-porous substrates (Figure 3b), when compared to that in flat-film substrates (Figure 3c).

**Figure 4:**
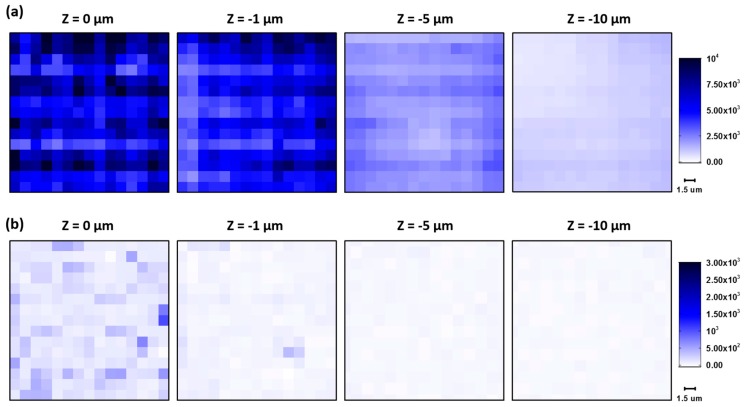
2D Raman mapping at four positions of the *z*-axis in (**a**) honeycomb-like SERS substrates and (**b**) flat-film SERS substrates. The concentration of analytes (R6G) is 10^−6^ M. The size of AuNPs is 67 nm.

Simultaneously, 2D Raman mapping at different positions of the *z*-axis was performed to analyze the depth-inductive effects at distinct vertical planes (20 μm × 20 μm) in the honeycomb-porous and flat-film substrates, as shown in Figure 4. With great focus on the strongest characteristic peak at 1510 cm^−1^, these images of 15 × 15 points (pixels) revealed the Raman intensity at each position. Raman mapping results also exhibited the significant enhancement of Raman signals in the honeycomb-porous substrates. As the scanning depth changed from 0 to −1 μm (ca. one layer of the pore), the collective Raman signals still remained intense in the honeycomb-porous substrates (Figure 4a). The enhanced intensity started to decrease as the scanning depth went deeper, and a distinct decrease was particularly observed at −5 μm. On the other hand, in flat-film substrates (Figure 4b), poor Raman signals were detected even on the surface, followed by a rapid drop as the scanning depth went deeper. There was virtually no Raman signal at −5 μm.

**Figure 5:**
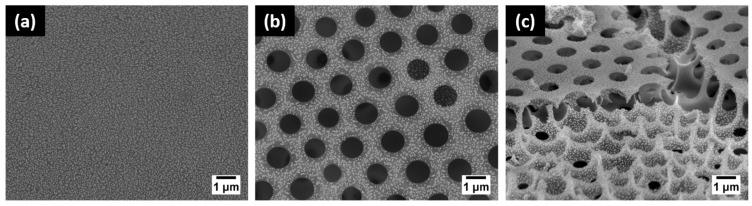
Top view of SEM images with AuNPs immobilized on (**a**) flat-film and (**b**) honeycomb-like SERS substrates; (**c**) cross-sectional image of honeycomb-like substrates. The size of AuNPs is 67 nm.

The morphologies of SERS substrates could be inspected with SEM images. In Figure 5a,b, large amounts of AuNPs were immobilized on both the flat films and honeycomb-patterned films. As shown in Figure 5c, the cross-sectional images of the honeycomb structure confirmed that plenty of AuNPs were adhered on the contours of the pores in the first layer of the honeycomb-porous structure (with the depth approximatly 1 μm beneath the surface). This attributed to the enormous Raman enhancement in the honeycomb-porous substrates, as indicated by the SERS mechanism in the multilayer honeycomb-porous substrates in Figure 3a.

**Figure 6:**
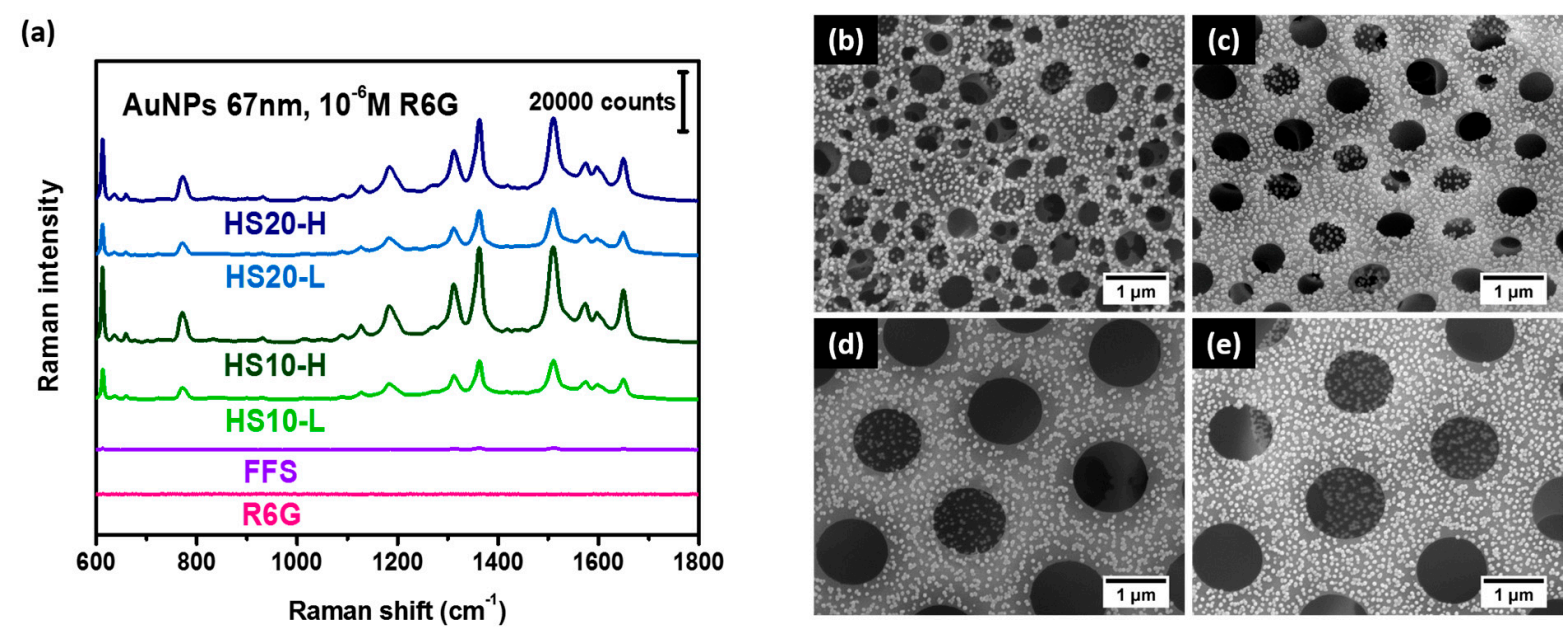
(**a**) Raman spectra of 10^−6^ M rhodamine 6G (R6G) in various honeycomb-like SERS substrates, flat-film substrates, and control (R6G); Top-viewed SEM images of SERS substrates with various polymer concentrations, and RH: (**b**) 10 mg/mL, 65% RH; (**c**) 20 mg/mL, 65% RH; (**d**) 10 mg/mL, 95% RH; (**e**) 20 mg/mL, 95% RH. The size of the AuNPs is 67 nm.

Moreover, the SERS detecting intensity affected by pore size and uniformity was discussed in Figure 6. By adjusting RH, polymer concentrations, and sizes of the AuNPs, various AuNPs/PU-PAZ SERS substrates were prepared for further SERS analysis. Figure 6a showed SERS spectra of R6G in different kinds of SERS substrates. When the honeycomb-porous substrates were fabricated at higher RH, stronger SERS intensities of R6G were observed, especially for the sample with 10 mg/mL of AuNPs/PU-PAZ SERS substrates. Figure 6b–e exhibited SEM images of four honeycomb-porous SERS substrates ((b) 10 mg/mL, 65% RH; (c) 20 mg/mL, 65% RH; (d) 10 mg/mL, 95% RH; (e) 20 mg/mL, 95% RH). Better uniformity of the honeycomb-porous structure could be achieved when the RH was high. However, relatively poor uniformity was obtained when the RH was low. This would induce certain fluctuations of SERS signals in different locations. In addition to investigating the sensitivity of HS10-H SERS substrates, surface enhancement factors (EF) were calculated via the following formula [66].

(1) $$\mathrm{EF}=\;\frac{I_{\mathrm{SERS}}/N_{\mathrm{SERS}}}{I_{\mathrm{Raman}}/N_{\mathrm{bulk}}}$$

where N_bulk_ is the number of analyte molecules (R6G) sampled in the bulk, and N_SERS_ is the number of R6G adsorbed on the SERS substrates. I_SERS_ and I_Raman_ denote the integrated intensities at specific peaks (1510 cm^−1^) in the SERS and Raman spectra. With the same spot size of the laser and the same content of R6G, the ratio of N_SERS_ to N_bulk_ could be deemed as the ratio of two concentrations of R6G. In other words, N_SERS_/N_bulk_ ratio is calculated as 10^−6^/10^−2^ which equals 10^−4^. The EF values in all of the honeycomb-like porous SERS substrates are higher than 3.3 × 10^5^. The strongest EF value is 9.2 × 10^5^ in the HS10-H SERS substrates. The EF value in the control group (flat film) is about 4.9 × 10^4^.

**Figure 7:**
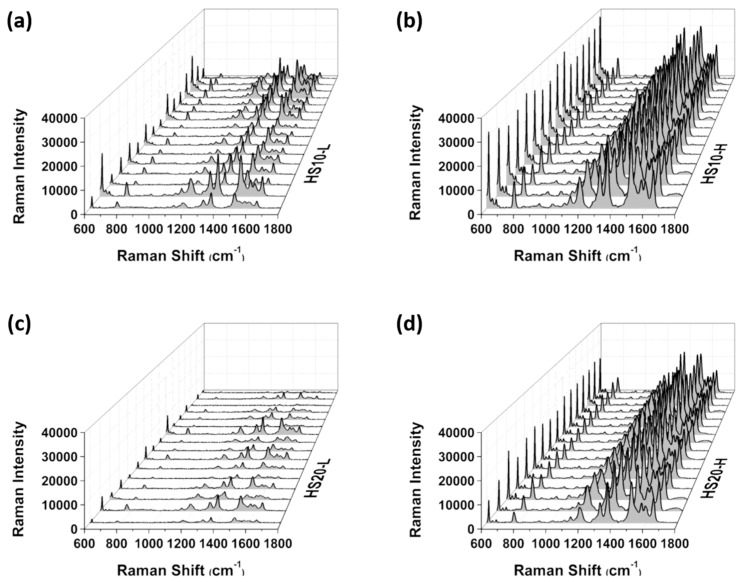
SERS spectra of 10^−6^ M R6G acquired from 16 randomly selected positions on various honeycomb-like SERS substrates: (**a**) 10 mg/mL, 65% RH; (**b**) 10 mg/mL, 95% RH; (**c**) 20 mg/mL, 65% RH; (**d**) 20 mg/mL, 95% RH. The size of the AuNPs is 67 nm.

Reproducibility and sensitivity of the SERS substrates is an important issue for assessing the performance of the substrates. All of the honeycomb-porous substrates not only outshined the flat-film substrates in SERS sensitivity, but also displayed good reproducibility, as shown in Figure 7. After statistical analysis based on Figure 7a–d, 10 mg/mL of PU-PAZ polymers at high RH (95%) exhibited the perfect reproducibility and sensitivity for SERS detection. It is important to note that the SERS substrates prepared under lower RH exhibited relatively poor reproducibility because of the presence of irregular arrays of honeycomb-like structures (Figure 7a,b).

**Figure 8:**
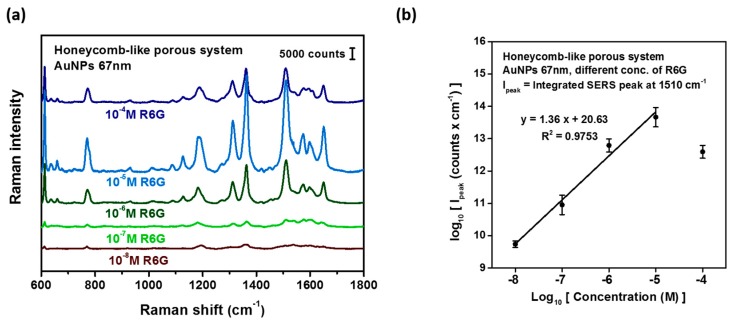
(**a**) SERS spectra of HS10-H SERS substrates (10 mg/mL PU-PAZ, 95% RH) with various concentrations of R6G molecules; (**b**) Limitation of detection (LOD) measurements in the HS10-H substrates with SERS signal at 1510 cm^−1^ as a function of the molecular concentration on a logarithmic scale. The size of the AuNPs is 67 nm.

Limitation of detection (LOD) experiments were conducted on the HS10-H SERS substrates immobilized with 67 nm of AuNPs using rhodamine 6G (R6G) as the detection analyte (Figure 8). The characterized peak at 1510 cm^−1^ was selected to be the integrated area for the LOD experiments. The results indicate that LOD would be 10^−8^ M. One might notice that the Raman intensity in the 10^−4^ M concentration of R6G was lower than that of the 10^−5^ M concentration of R6G. This is because the R6G molecules capable of exhibiting laser-induced fluorescence were condensed to aggregate and form a crystallization of R6G molecules in a 10^−4^ M concentration of R6G. Therefore, when spotted by the laser, the position of the crystallized R6G generated intense fluorescence which would shield the SERS signals. In addition, the crystallization of R6G may affect the Raman scattering and cause certain difficulty in SERS detection.

**Figure 9:**
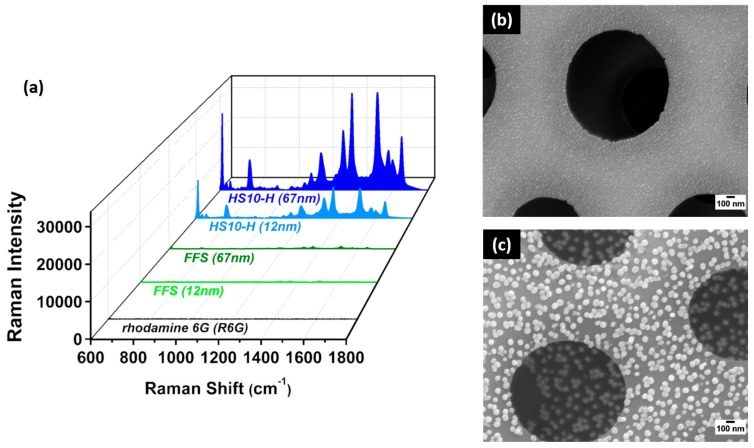
(**a**) SERS spectra of R6G (10^−6^ M), acquired from HS10-Hs and flat film substrates (FFSs) immobilized with AuNPs; SEM images of HS10-Hs immobilized with (**b**) 12 nm (**c**) 67 nm AuNPs. The control was 10^−6^ M of R6G.

**Figure 10:**
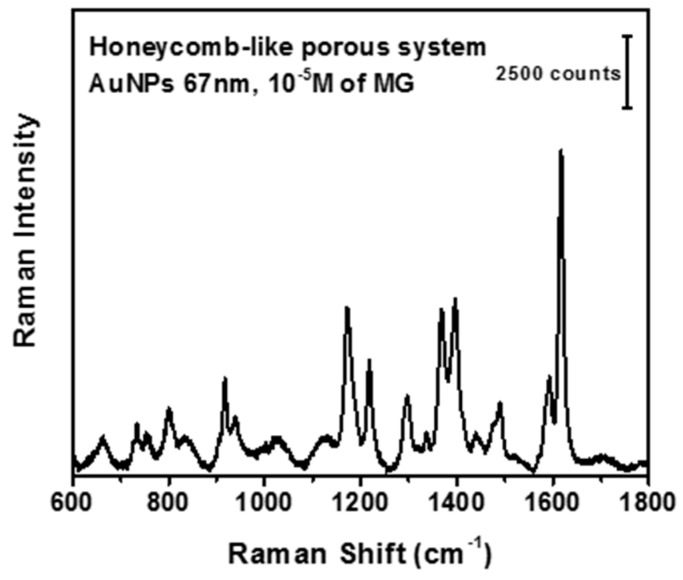
SERS spectra of malachite green (10^−5^ M) in the application of water pollutants detection.

It is reported that the optimum size of metal nanoparticles for maximum SERS enhancement was in the range between 30 and 80 nm [1,67]. Therefore, we investigated the SERS intensity for the substrates immobilized with different AuNPs sizes (12 and 67 nm), as shown in Figure 9. Although both substrates adsorbed plenty of AuNPs on the surface, the SERS intensity of the substrate immobilized with 67 nm AuNPs was superior to that of the substrate immobilized with 12 nm AuNPs, as shown in Figure 9a. In this case, the larger sizes of AuNPs could provide a stronger surface plasmon resonance effect during the laser irradiation, when compared with the smaller ones. Therefore, the optimal SERS substrate was found for the HS10-H substrate immobilized with 67 nm AuNPs, showing excellent sensitivity and reproducibility. Figure 9b,c indicates the morphologies of the SERS substrates immobilized with two sizes of AuNPs by SEM observation. In addition, water pollutants (toxicities) detection of our SERS substrates was performed by detecting trace-levels of malachite green (10^−5^ M), which would be contracted by fishes and pose a significant health risk to human beings (Figure 10). This demonstrated the valid applicability of our SERS substrates.

## 4. Conclusions

In this study, we report SERS-active substrates with high sensitivity and reproducibility via hot-spots resonance effects based on the AuNPs/PU-PAZ honeycomb-porous substrates. Through the BF process, highly ordered structures with multilayers of porosity were acquired. With the immobilization of AuNPs (67 nm) onto honeycomb-like polymeric films prepared at the polymer concentration of 10 mg/mL under 95% RH, the optimal SERS substrates were developed. The immobilized honeycomb-like polymeric films demonstrated significant SERS intensity and good reproducibility due to the presence of multilayered honeycomb-like porous structure, when compared to the flat-film substrates. Moreover, we explored a SERS mechanism of hot-spots effects in 3D honeycomb-like polymeric films using 2D Raman mapping in the *z*-axis. In a nutshell, these stable SERS-active substrates with the characteristics of facile and low-cost preparation provide great potential in detecting trace-level and diverse molecules, such as for the application of water pollutants (malachite green, 10^−5^ M) detection.

## Figures and Tables

**Table 1:** Pore sizes of honeycomb-like and flat-film substrates.

Sample code	PU-PAZ conc. (mg/mL)	RH (%)	Pore size (μm)
HS5-L	5	65	0.39 ± 0.08
HS5-H	5	95	0.73 ± 0.19
HS10-L	10	65	0.83 ± 0.14
HS10-H	10	95	1.46 ± 0.09
HS20-L	20	65	0.61 ± 0.10
HS20-H	20	95	1.24 ± 0.43
FFS ^1^	20	-	-

^1^ Flat-film substrate.
